# *GLI1*-altered mesenchymal tumor involving the parietal pleura: case report and literature review

**DOI:** 10.3389/fonc.2025.1484206

**Published:** 2025-02-10

**Authors:** Yuanli Zhong, Baizhou Li, Gangping Wang, Yuqing Liu, Zhenwei Chen

**Affiliations:** Department of Pathology, The Fourth Affiliated Hospital of School of Medicine, and International School of Medicine, International Institutes of Medicine, Zhejiang University, Yiwu, Zhejiang, China

**Keywords:** *GLI1*, mesenchymal tumor, *PTCH1-GLI1* fusion, parietal pleura, malignant potential, risk factors

## Abstract

*GLI1*-altered mesenchymal tumors represent a rare category of soft tissue tumors that have recently been incorporated into the classification of head and neck soft tissue tumors in the fifth edition of the World Health Organization (WHO) classification. However, their precise nature remains undefined, and they have yet to be assigned an ICD code. These tumors are predominantly located in the head and neck region and display distinctive pathological morphology and molecular characteristics. We present the first documented case of a *GLI1*-altered mesenchymal tumor occurring in the pleura. Microscopic examination revealed that the tumor was composed of ovoid-to-round and vaguely epithelioid cells, as well as a few spindle cells, all exhibiting a uniform morphology and organized in a nested and reticular arrangement, accompanied by a rich capillary network in the stroma. Immunohistochemical staining demonstrated positivity for CD56, S-100, and SMA. Next-generation sequencing (NGS) revealed a *PTCH1-GLI1* fusion. Based on the morphological and immunophenotypic characteristics, molecular studies confirmed the diagnosis of a *GLI1*-altered mesenchymal tumor. At the 15-month follow-up, the patient was alive. We conducted a review of all cases of recurrence and metastasis, concluding that this type of tumor has a distinct propensity to metastasize to the lungs. The tumor exhibits malignant potential, and factors such as its occurrence outside the head and neck region, high-grade histological morphology, active mitosis (>5/10HPF), necrosis and *PTCH1-GLI1* fusion are all considered potential risk factors.

## Introduction

In 2004, Dahlen et al. collected a unique cohort of soft tissue tumors located in the tongue (3 cases), stomach (1 case), and lower leg (1 case). These tumors were morphologically characterized by a lobulated structure composed of oval and short spindle cells, enriched with a network of thin-walled vessels. Immunohistochemical analysis revealed positivity for SMA and Laminin, while electron microscopy observations indicated differentiation features consistent with pericytes. Molecular results demonstrated the presence of the *ACTB::GLI1* fusion. No recurrence or metastasis was observed during a median follow-up period of 24 months. Based on these findings, the researchers classified this group of tumors as benign tumors within the lineage of perivascular cell tumors and designated them as “Pericytoma with t(7;12)” (1). Subsequently, additional reports emerged documenting cases at various anatomical sites and identifying different *GLI1* fusion partner genes, with some cases exhibiting recurrence and metastasis. In the fifth edition of the WHO Classification of Head and Neck Tumors, this tumor type was included and renamed *GLI1*-altered mesenchymal tumors. Due to its broad morphological and immunohistochemical spectrum and the absence of a clear differentiation pathway, it is currently classified as a tumor with uncertain differentiation. This report presents the first documented case of a *GLI1*-altered mesenchymal tumor located in the pleura (2).

## Case presentation

**Figure 1:**
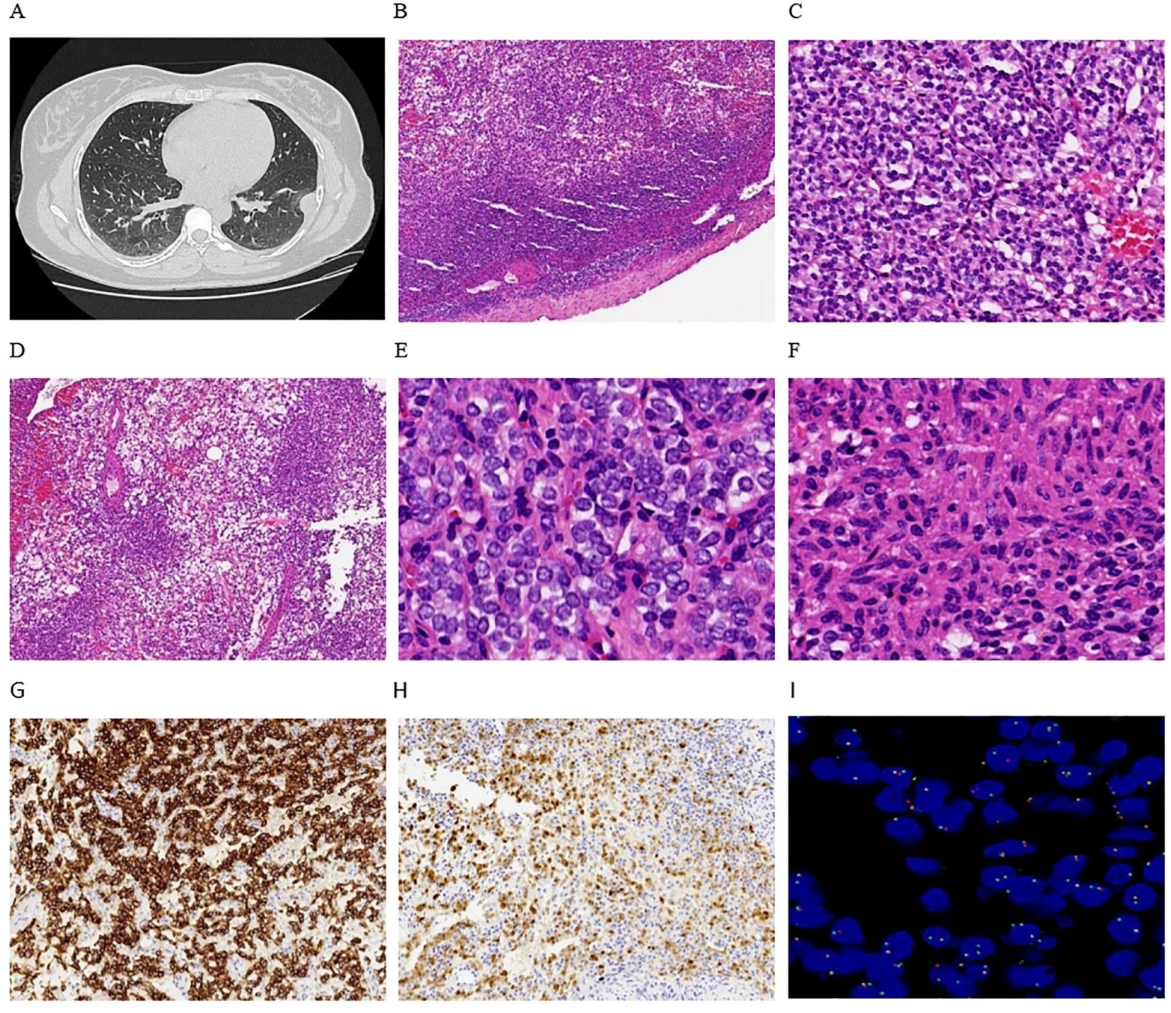
Clinical and pathological findings. **(A)** Chest CT revealed a nodule on the left parietal pleura with well-defined boundaries. **(B)** Microscopically, the tumor also exhibited clear boundaries, with mesothelial cells visible at the periphery (4x). **(C)** The tumor cells were arranged in a nested pattern (10x). **(D)** Reticulated pattern (4x). **(E)** Monomorphic round cells (40x). **(F)** Focal spindling (40x). **(G)** Tumors are immunoreactive for CD56 (10x). **(H)** Focal expression of S-100 (10x). **(I)** FISH revealed positive *GLI1* gene break-apart signals, primarily manifesting as single green atypical signals (100x).

The patient is a 34-year-old female who was admitted to the thoracic surgery department of our hospital on September 17, 2023, after the detection of a pleural nodule during a physical examination conducted more than a year earlier. During her hospitalization, a chest CT scan revealed a well-defined nodule in the left pleura, measuring approximately 22 mm in length, which was likely of pleural origin (**Figure 1A**). After ruling out any surgical contraindications, a resection of the left pleural nodule was performed on September 22, 2023.

**Figure 2:**
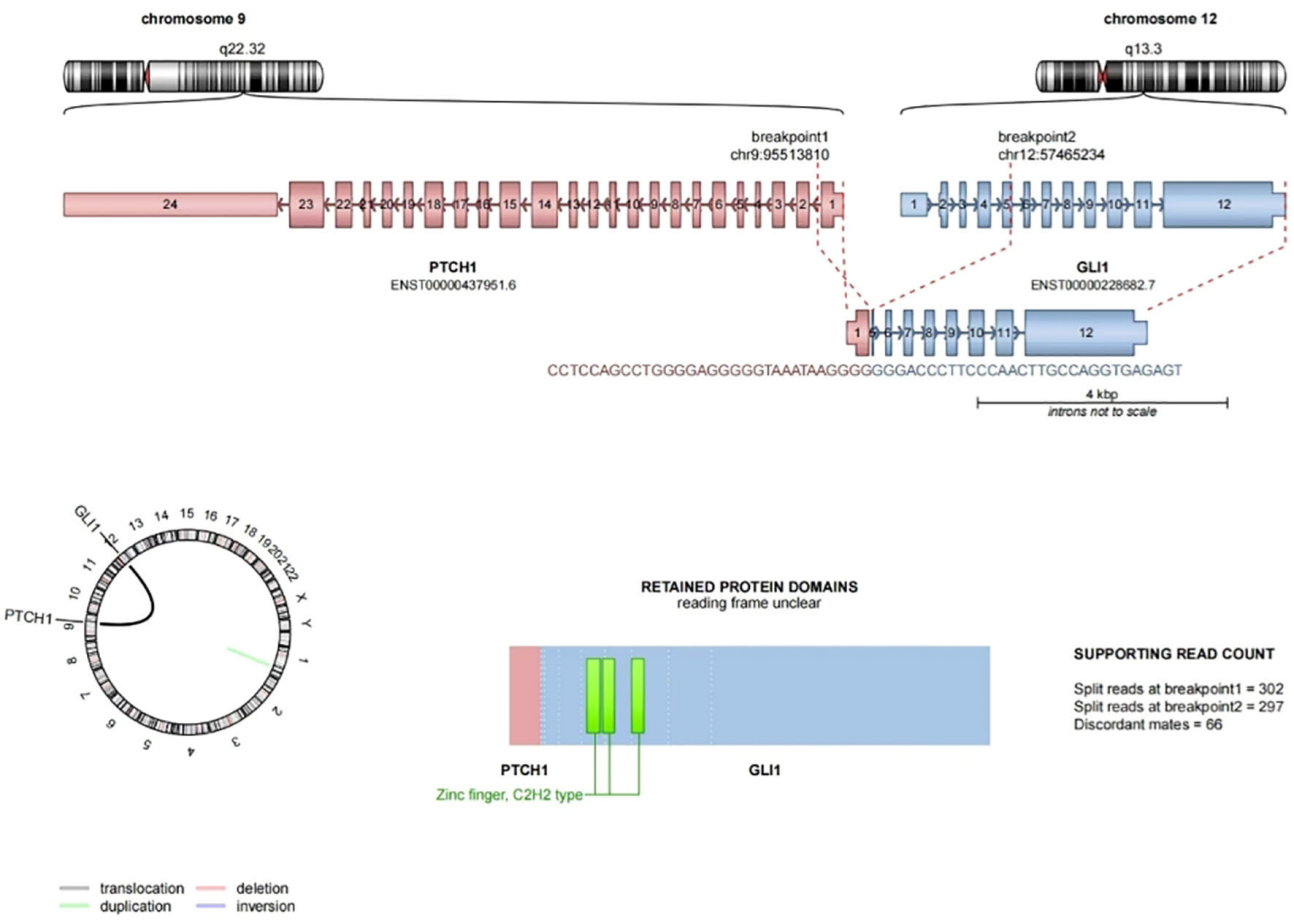
*PTCH1-GLI1* fusion sequence-structure characterization. Exon 1 of the *PTCH1* gene is fused to exon 5 of *GLI1*, GLI1 part of the fusion protein retains its FOXP coiled-coil domain (FOXP-CC) (the bottom right is not shown) and three zinc fingers of C2H2 type (C2H2 Zn fingers).

Pathological examination revealed a nodular object measuring 25×18×15 mm, characterized as solid cystic with a gray-red coloration. Microscopic observation showed clearly defined tumor boundaries (**Figure 1B**), with the fibrocystic wall tissue and solid areas displaying nested (**Figure 1C**), reticulated (**Figure 1D**), and nodular patterns. The tumor cells were composed of monomorphic ovoid-to-round and vaguely epithelioid cells (**Figure 1E**), as well as a few spindle cells (**Figure 1F**). The cytoplasm ranged from pale-eosinophilic to clear. The nuclei were round to oval, with fine chromatin, and the nucleoli were either inconspicuous or small, with occasional mitotic figures observed (< 1/2 mm²). The tumor cells were interspersed within a well-developed arborizing capillary network, and no significant necrosis was detected. Immunohistochemical staining results showed diffuse positivity for CD56 (**Figure 1G**) and focal positivity for S-100 (**Figure 1H**) and SMA, while AE1/AE3, Synaptophysin, Chromogranin A, CD34, Desmin, Calretinin, WT-1, and TTF1 all yielded negative results. The Ki-67 labeling index was approximately 2%. FISH analysis revealed *GLI1* gene break-apart signals, indicating positive evidence for *GLI1* rearrangement, primarily presenting as single green atypical signals (**Figure 1I**); the abnormal *GLI1* signals were present in 60% of the cells. Targeted RNA sequencing revealed the presence of a *PTCH1-GLI1* fusion (**Figure 2**). The identified fusion in this case consists of the 3′ end of exon 1 (5′UTR region) of PTCH1 and the 5′ end of exon 5 of GLI1, preserving the FOXP coiled-coil domain (FOXP-CC) as well as the DNA-binding zinc finger domains (C2H2 Zn fingers) of GLI1. These morphological and molecular findings support the diagnosis of a *GLI1*-altered (*PTCH1-GLI1* fusion) mesenchymal tumor. During a 15-month follow-up time, the patient did not develop recurrent and metastatic disease.

## Discussion

*GLI1*-altered mesenchymal tumors represent a rare category of neoplasms, with only 90 cases documented in the English literature to date (**Supplementary Table**). These tumors predominantly affect young and middle-aged adults, with a median age of onset at 40 years, and they exhibit no significant gender predisposition. Approximately 33% (30/90 cases) of the lesions occurred in the head and neck region, followed by the limbs, especially the thighs, which was basically consistent with previous studies (3–5). Less common sites include the stomach (1, 6), intestines (7, 8), ovaries (9, 10), uterus (11, 12), lungs (13), kidneys (11), and bone (14, 15). In this report, we present the first documented case of a *GLI1*-altered mesenchymal tumor arising in the pleura. The clinical manifestations of these tumors vary by location, with the majority of cases exhibiting slow and painless growth.

*GLI1*-altered mesenchymal tumors display complex and variable morphological structures and cytological features. These tumors typically present as lobulated formations, with tumor cells arranged in sheets, nests, and occasionally in a sieve-like, reticular, microcystic, pseudoglandular, papillary and fascicular patterns (16). The cellular morphology is relatively uniform, primarily characterized by epithelioid, round, oval, or spindle. The cytoplasm can be eosinophilic or clear, while the cell nuclei are round, featuring fine chromatin and small, pinpoint nucleoli. In some instances, high-grade morphology is observed, which is characterized by pleomorphic spindle cells and focal epithelioid cells (12). The number of nuclear divisions varies from 0 to 5 per mm², with a few cases exhibiting counts as high as 40 per 10 high-power fields (HPF) (12, 17). The protrusion of tumor cells into the vascular space is a common phenomenon. Additionally, there are rich dendritic vascular networks present in the stroma, and in certain cases, mesenchymal mucosis or hyalinosis may be observed (4, 5). Rare instances of necrosis have also been noted (18). The case presented demonstrates a typical histological structure, cellular characteristics, and a rich stroma of dendritic vascular networks. *GLI1*-altered mesenchymal tumors exhibit non-specific immunohistochemical characteristics. Most cases demonstrate expression of CD56, while S-100 expression varies, and some cases also express SMA, AE1/AE3, CD10, cyclin D1, and BCOR (3, 11). In instances of *GLI1* amplification, the expression of MDM2, CDK4, and STAT6 may also be detected (13). Other markers, such as CD34, ERG, Synaptophysin, Chromogranin A, desmin, calponin, and SOX10, are consistently negative. The positivity for CD56, S-100, and SMA in this case aligns with findings from previous studies. Notably, the immunohistochemical phenotype lacks specificity; therefore, reliance on positive immune responses to these relatively non-specific markers may result in misdiagnosis. Parrak et al. reported that GLI1 immunohistochemical expression has a sensitivity of 91% and a specificity of 98% for diagnosing *GLI1*-altered mesenchymal tumors. GLI1 expression is localized in the cytoplasm and nucleus or may be confined solely to the nucleus (19). In the absence of *GLI1* molecular testing, GLI1 immunohistochemistry can serve as a valuable diagnostic adjunct, aiding in the differentiation of *GLI1*-altered mesenchymal tumors.

Molecular events associated with *GLI1*-altered mesenchymal tumors include *GLI1* gene fusion and amplification. A total of 76% (68/90 cases) were classified as fusion, which included one case of both amplification and fusion; however, the specific fusion site remained unclear (3). In the remaining three cases, GLI1 fusion was identified through FISH, although the fusion partner was not determined. Notably, sixty-six percent (42/64 cases) of the identified fusion partners were *ACTB*, followed by *MALAT1, PTCH1*, and several rarer partners such as *APOD, DERA, SYT, NCOR2, PAMR1, DDIT3, KDM2B* and *TUBAIA* (3, 20). Occasionally, GLI1 can also be fused with two genes simultaneously (21). These various fusion genes retain the FOXP coiled-coil domain and the DNA-binding zinc finger domain located at the 3’ end of the *GLI1* gene. However, the promoter at the 5’ end of the *GLI1* gene is replaced by the highly active promoter of the fusion partner, which results in elevated expression of *GLI1* and subsequent activation of downstream gene transcription, thereby promoting tumor development (22, 23). Notably, the fusion of *GLI1* with different partner genes yields similar pathological morphology, immunohistochemical profiles (3). Additionally, approximately 26% (23/90 cases) demonstrated *GLI1* gene amplification, often accompanied by the co-amplification of genes located near the 12q13-15 locus, including *DDIT3, CDK4, MDM2, STAT6*, and *HMGA2*. This amplification can lead to the overexpression of the corresponding proteins, which can be identified through immunohistochemical methods (13). In this case, *GLI1* gene break-apart signals were successfully detected using FISH technology. NGS revealed a *PTCH1-GLI1* fusion and the combined morphology consistently pointed to *GLI1*-altered mesenchymal tumor.

Surgery serves as the primary treatment for *GLI1*-altered mesenchymal tumors, while inhibitors of the sonic hedgehog signaling pathway may offer potential targeted therapy for tumors exhibiting *GLI1* activation (24, 25). Most *GLI1*-altered mesenchymal tumors demonstrate an indolent clinical course following complete surgical resection. In this case, the patient remained stable at 15 months post-surgery. Due to the limited number of cases, the 5th edition of the WHO classification for Head and Neck tumors does not provide clear indications regarding biological behavior, nor is there an associated ICD coding. We have compiled data from the English literature concerning *GLI1*-altered mesenchymal tumors that have experienced recurrence and metastasis (**Table 1**).

**Table 1:** Summary of Recurrent and Metastatic GLI1-Altered Mesenchymal Tumors.

	Age/Sex	Site	Outcome (FU) (mo)	Pathologic Features	Mitoses	Necrosis	*GLI1* Alteration
Antonescu et al	34/F	Neck	LR, mets to LN and lung. AWD (80)	Round to epithelioid, myxoid stroma	1-5/10HPF	focal	*PTCH1-GLI1*
	30/F	Foot	LR, Inguinal LN met. AWD (21)	Round to epithelioid	1-5/10HPF	No	*ACTB-GLI1*
	79/F	Retroperitoneum	Inguinal LN mets	Round to epithelioid	1-5/10HPF	No	*ACTB-GLI1*
Agaram et al	39/M	Neck	LR, lung met. AWD (26)	Prominent spindle cell	>25/10HPF	Yes	*GLI1* Amp
	51/F	Back	LR (16)	Round to epithelioid, focally increased atypia	>25/10HPF	Yes	*GLI1* Amp
KerrDA et al	57/F	Tibia	Rib met, AWD (27)	Round to epithelioid	NA	NA	*ACTB-GLI1*
	62/M	Scapula	Lung (84) and soft tissue/bone (180) met, AWD	Round to epithelioid	NA	NA	*ACTB-GLI1*
Prall OWJ et al	73/M	Jejunum	Multiple mets, AWD (312)	Epithelioid and spindled	30/2mm^2^	focal	*MALAT-GLI1*
Alwaqf RR et al	54/F	Ovary	Colonic mesentery met, AWD (49)	Epithelioid	39/10HPF	Yes	*PTCH1-GLI1*
Liu J et al	8/M	Mouth floor	LR (27 and 40)	Round to epithelioid	NA	No	*ACTB-GLI1*
	1.3/M	Elbow	LR (4)	Spindled to ovoid	NA	No	*GLI1* Amp and break-apart
Zhong H et al	56/M	Lingual	mets to LN and sacral (27). AWD (36)	Round to epithelioid, myxoedematous stroma	5/10HPF	focal	*GLI1* gene break-apart Signals (FISH)
Argani P et al	33/F	Renal pelvis	LR (25)	Ovoid-spindle cells	<1/10HPF	No	*GLI1* gene break-apart Signals (FISH)
	49/F	Uterine	LR (24) and mets to brain (29). DOD (36)	Round to epithelioid (met) and spindled (LR)	16/10HPF	Yes	*GLI1* Amp
	88/F	Uterine	involving the rectal adventitia	Ovoid to spindle cell, myxoid stroma	15/10HPF	focal	*GLI1* Amp
Punjabi LS et al	57/F	Uterine	LR, mets to LN and lung (11). DOD (18)	Sarcomatous, pleomorphic cell	40/10HPF	NA	*PAMR1-GLI1*
Machado I et al	65/M	Left knee subcutaneous	skin of left tibial (1). NED (8)	Ovoid to epithelioid and spindled, microcystic	2/10HPF	focal	*GLI1* Amp
	66/M	chest	Recurrence; NED(1)	Ovoid cells, focal pleomorphism	8/10HPF	No	GLI1 amp
Kerr DA et al	40/F	Right thigh	bone and soft tissue met (77). NED (89)	Ovoid to epithelioid cells, myxohyaline stroma	6/2mm^2^	No	*ACTB-GLI1*
	71/M	NA	lung met (bilateral). AWD (16)	Ovoid to epithelioid cells, myxoid stroma	1/2mm^2^	No	*ACTB-GLI1*
	31/M	Right leg	bone met (12), lung met (54). AWD (54)	Ovoid to epithelioid cells, myxoidto collagenous stroma	6/2mm^2^	No	*ACTB-GLI1*
	56/M	Thoracic vertebra T9	LR (18). AWD (21)	Ovoid to epithelioid cells, myxoid to collagenous stroma	<1/2mm^2^	No	*ACTB-GLI1*
	26/F	T8-T9 Epidural tumor	LR (12, 105), lung met (36, 105). AWD (108)	Ovoid cells, myxoid stroma	2/2mm^2^	No	*ACTB-GLI1*
	46/M	Right pelvis soft tissue	LR	Ovoid to round cells, solid to sieve-like, myxoid stroma	1/2mm^2^	No	*ACTB-GLI1*
	30/M	NA	lung met	Ovoid to epithelioid cells, pseudoglandular, myxoid matrix	<1/2mm^2^	No	*ACTB-GLI1*
	37/F	Right neck	LR	Ovoid to epithelioid cells, corded, myxoid stroma	2/2mm^2^	No	*PTCH1-GLI1*
Yajuan J. Liu	35/M	chest wall (multiple)	regional metastasis (involve a lymph node); NED(10)	ovoid to spindled cells	Rare	No	*TUBA1A-GLI1*

F, female; M, male; NA, not available; FU, follow up; mo, months; LR, local recurrence; LN, lymph node; met, metastasis; AWD, alive with disease; NED, no evidence; Amp, amplification.

In a cohort of 64 patients with follow-up data, 42% (27/64 cases) experienced recurrence and metastasis. Among these cases, 20% (13/64 cases) exhibited local recurrence, while 30% (19/64 cases) developed distant metastasis. Notably, of the patients with distant metastasis, 47% (9/19 cases) had metastasized to the lungs, including 2 patients who presented with bilateral lung metastases (7, 18). Soft tissue tumors typically metastasize through the bloodstream, and the lungs, characterized by an extensive capillary bed, provide a conducive environment for circulating tumor cells to stagnate. Furthermore, fibroblasts within the lungs may facilitate the extravasation and implantation of these circulating tumor cells by reshaping the local immune microenvironment (26). *GLI1*-altered mesenchymal tumors are distinguished by the protrusion of tumor cells into the vascular space, which, along with the aforementioned factors, contributes significantly to their propensity to metastasize to the lungs. Additionally, these tumors may also metastasize to lymph nodes, bone (27), liver (7), brain, intestines (11), and various soft tissue sites (28). The average age of recurrence and metastasis was 47 years, with no significant difference observed between males and females. Approximately 80% (20/25 cases) of the primary tumors were located outside the head and neck region, while the primary sites for 2 patients with distant metastasis could not be determined. Microscopically, the tumor cells were predominantly oval to epithelioid, arranged in nests with interstitial mucosis, and the number of mitotic figures varied. Among the patients with available prognostic information, 12 exhibited more than 5 mitotic images per 10 HPF, of which 83% (10/12 cases) experienced relapses and metastases. Among the 15 patients with necrosis present within the tumor, up to 67% (10/15 cases) developed recurrence and metastasis. At the molecular level, we observed that 50% (3/6 cases) of *PTCH1-GLI1* tumors recurred and metastasized, while the remaining two cases had a follow-up period of less than four months, which is insufficient to reliably assess prognosis. Among the cases that recurred and metastasized, one exhibited bland morphology with unremarkable nuclear division and necrosis, yet still experienced a recurrence. This finding suggests that alterations in the *PTCH1-GLI1* gene may be associated with a poor prognosis. Although the recurrence and metastasis rate was as high as 42%, the mortality rate was only 3% (2/64 cases), with both fatal cases occurring in the uterus. The tumor exhibits active mitotic figures, and one case demonstrates a high-grade sarcomatoid histological morphology. These findings suggest that this tumor possesses aggressive biological potential, and it may be more appropriate to classify it as a *GLI1*-altered mesenchymal tumor with malignant potential. The external location of the tumor relative to the head and neck, along with high-grade histological morphology, active mitosis (>5/10HPF), necrosis, and *PTCH1-GLI1* gene alteration are all significant risk factors for recurrence and metastasis. Among these factors, high-grade histological morphology is the most aggressive. The presence of such morphology indicates that the tumor can be classified as malignant. Additionally, there was one case that experienced a recurrence without having any of the aforementioned risk factors. This case is situated outside the head and neck region, presenting a risk of recurrence and metastasis; therefore, stringent follow-up observation is essential. The statistical analysis of prognostic data is complicated by the variability in follow-up periods, as some cases have a brief follow-up duration while others extend beyond ten years before recurrence or metastasis occurs. This disparity may introduce bias into the data, thereby compromising its accuracy. To achieve a more precise assessment of prognosis, a larger sample size and an extended follow-up period are imperative. In our study, both tumor location and molecular changes are identified as risk factors, and we will conduct close follow-up.

The differential diagnosis includes: (1) Mesothelioma: This case occurred in the pleura, with chest CT suggesting a possible pleural origin. Classic epithelioid mesothelioma is characterized by papillary, trabecular, and solid arrangements, exhibiting marked cellular atypia and pleomorphism. It is positive for mesothelial markers in immunohistochemistry, including Calretinin, WT1, and D2-40. (2) Glomus tumor: Glomus tumors share morphological similarities with *GLI1*-altered mesenchymal tumors, but they diffusely express SMA and Caldesmon in histochemistry, with molecular alterations characterized by *NOTCH* gene fusion. (3) Neuroendocrine tumor: Comprised of morphologically uniform cells with rich stromal vasculature, these tumors can be confused with neuroendocrine tumors. Differentiation can be achieved through immunohistochemistry, as neuroendocrine tumors characteristically exhibit diffuse positivity for neuroendocrine markers such as CD56, Synaptophysin, and Chromogranin A. (4) Ectomesenchymal chondromyxoid tumor: This tumor typically occurs on the dorsal side of the tongue and exhibits morphology similar to *GLI1*-altered mesenchymal tumors characterized by interstitial mucinous change. Notably, tumor cells generally express GFAP and S-100, with the molecular alteration identified as the *RREB1-MKL2* fusion. (5) Other *GLI1* gene-altered tumors include gastroblastoma, gastric plexiform fibromyxoma, and liposarcoma, each of which presents distinct morphological and histochemical features.

## Conclusion

*GLI1*-altered mesenchymal tumors are rare neoplasms characterized by distinct morphological and molecular features, primarily found in the head and neck, although they can also occur in other anatomical locations. These tumors are associated with a significant risk of recurrence and metastasis; even those with benign cellular characteristics may exhibit metastatic behavior, particularly to the lungs. Key risk factors for recurrence and metastasis include tumor location outside the head and neck, high-grade histological morphology, active mitosis (>5/10HPF), necrosis, and *PTCH1-GLI1* gene alteration. Notably, high-grade histological morphology correlates with the most aggressive behavior of these tumors. As a result, they possess a considerable potential for malignancy and may sometimes exhibit malignant biological characteristics. Therefore, close follow-up is essential, making accurate diagnosis and continuous monitoring of these tumors critically important.

## Data Availability

The original contributions presented in the study are included in the article/**Supplementary Material**. Further inquiries can be directed to the corresponding author.
