# Supplementary material for: GLI1-altered mesenchymal tumor involving the parietal pleura: case report and literature review
Source: Front Oncol. 2025 Feb 10;15:1484206. doi: 10.3389/fonc.2025.1484206 (PMC11847683; doi:10.3389/fonc.2025.1484206)
Supplement: Supplementary file 1 [file DataSheet1.docx]

| **Supplementary table Summary of *GLI1*-Altered Mesenchymal Tumors** | | | | | | | |
| --- | --- | --- | --- | --- | --- | --- | --- |
|  | Age/Sex | Site | Outcome (FU) (mo) | Pathologic Features | MI | Necrosis | GLI1 Alteration |
| Dahlén A et al | 61/M | Calf | NED(24) | Spindle-to-ovoid | ＜1/10HPF | No | *ACTB-GLI1* |
|  | 27/F | Tongue | NED(60) | Spindle-to-ovoid | ＜1/10HPF | No | *ACTB-GLI1* |
|  | 11/M | Tongue | NED(22) | Spindle-to-ovoid | ＜1/10HPF | No | *ACTB-GLI1* |
|  | 65/F | Stomach | NED(24) | Spindle-to-ovoid | ＜1/10HPF | No | *ACTB-GLI1* |
|  | 12/F | Tongue | NED(120) | Spindle-to-ovoid | ＜1/10HPF | No | *ACTB-GLI1* |
| Bridge JA et al | 67/M | Talus | NED(6) | Spindle-to-ovoid, focally myxoid | infrequent | NA | *ACTB-GLI1* |
| Antonescu et al | 34/F | Neck | LR, mets to LN and lung AWD 80 mo | Round to epithelioid myxoid stroma | 1-5/10HPF | focal | *PTCH1-GLI1* |
|  | 30/F | Foot | LR* Inguinal LN met 21 mo AWD | Round to epithelioid | 1-5/10HPF | No | *ACTB-GLI1* |
|  | 79/F | retroperitoneum; | Inguinal LN mets | Round to epithelioid | 1-5/10HPF | No | *ACTB-GLI1* |
|  | 20/M | Thigh | NA | Round to epithelioid | 1-5/10HPF | No | *ACTB-GLI1* |
|  | 16/M | C2 spine | Recent case | Round to epithelioid | 1-5/10HPF | No | *MALAT-GLI1* |
|  | 38/F | Chest wall | Recent case | Round to epithelioid | 1-5/10HPF | No | *ACTB-GLI1* |
| Koh NWC et al | 11/F | Ovary | Recent case | Round to spindled, myxoid | rare | Yes | *ACTB-GLI1* |
| Agaram et al | 39/M | Neck | LR/DM(lung)/AWD (26) | prominent spindle cell | >25/10HPF | Yes | *GLI1* Amp, |
|  | 51/F | Back | LR(16) | Round to epithelioid, focally increased atypia | >25/10HPF | Yes | *GLI1* Amp |
|  | 4/F | Shoulder | NA | ovoid | 5/10HPF | No | *GLI1* Amp |
|  | 10/M | Finger | NA | Round to epithelioid | 15/10HPF | No | *GLI1* Amp |
|  | 17/M | Thigh | NA | Round to epithelioid, myxoid | 5/10HPF | No | *GLI1* Amp |
|  | 23/F | Thigh | NED(36) | Round to epithelioid | 2/10HPF | No | *GLI1* Amp |
|  | 26/M | Lung | NA | Round to epithelioid | 4/10HPF | No | *GLI1* Amp |
|  | 54/F | Elbow | NA | Round to epithelioid | 15/10HPF | Yes | *GLI1* Amp |
|  | 60/M | Forearm | NA | Round to epithelioid | 10/10HPF | No | *GLI1* Amp |
|  | 65/M | Tongue | NA | Round to epithelioid | 5/10HPF | No | *GLI1* Amp |
| KerrDA et al. | 57/F | Tibia | Rib met, 27, AWD | Round to epithelioid | 5/10HPF | No | *ACTB-GLI1* |
|  | 62/M | Scapula | Lung (84) and soft tissue/bone (180) met, AWD | Round to epithelioid | 1/50HPF | No | *ACTB-GLI1* |
|  | 41/F | Ovary | NED(14) | ovoid | 1/50HPF | focal | *ACTB-GLI1* |
| Xu B et al | 46/F | Tongue | NED(3) | Round to epithelioid | 0/10HPF | No | *GLI1* Amp |
|  | 60/M | Tongue | NA | Round to epithelioid | 11/10HPF | No | *GLI1* Amp |
|  | 38/M | Tongue | NED(2) | Round to epithelioid | 1/10HPF | No | *PTCH1-GLI1* |
|  | 37/M | Neck | NED(30) | Round to epithelioid | 0/10HPF | No | *ACTB-GLI1* |
|  | 1/M | Tongue | NED(2) | Round to epithelioid | 4/10HPF | No | *ACTB-GLI1* |
|  | 28/M | Tongue | NA | Round to epithelioid | 0/10HPF | No | *ACTB-GLI1* |
|  | 14/M | Tongue | NA | Round to epithelioid | 0/10HPF | No | *ACTB-GLI1* |
|  | 56/F | Tongue | NA | Round to epithelioid | 8/10HPF | No | *MALAT-GLI1* |
| Panagopoulos I et al | 83/F | Thigh | NA | Ovoid | ? | ? | *ACTB-GLI1* |
| Prall OWJ et al | 73/M | Jejunum  (4.5) | Multiple mets, AWD 312 | Epithelioid and spindled | 30/2mm^2^ | focal | *MALAT-GLI1* |
| Aivazian K et al | 9/F | Great toe | NED(10) | Round to epithelioid, cribriform gland-like structures | 8/mm^2^ | No | *GLI1* Amp |
| Lopez-Nunez O et al | 40/F | Thigh | NA | Round to epithelioid, myxoid | 4/10HPF | No | *APOD-GLI1* |
| Nitta Y et al | 11/F | Shoulder | NED(24) | Short spindled | 3/10HPF | No | *DERA-GLI1* |
| Alwaqf RR et al | 54/F | Ovary | Colonic mesentery met, AWD 49 | Epithelioid | 39/10HPF | Yes | *PTCH1-GLI1* |
| Benjamin T. Rollins | 69/M | left buttock | NED(6) | round-to-ovoid cells | 0/10HPF | Focal | *ACTB-GLI1* |
| Liu JH et al | 8/M | Mouth floor | LR(27 and 40) | Round to epithelioid | NA | No | *ACTB-GLI1* |
|  | 1.3/M | Elbow | LR(4) | Spindled to ovoid | NA | No | *GLI1* Amp and break-apart |
|  | 48/F | Stomach | NED(18) | Round to epithelioid, focally spindled | NA | Yes | *ACTB-GLI1* |
|  | 27/M | Tongue base | NED(16) | Round to epithelioid | NA | No | *ACTB-GLI1* |
|  | 52/F | Uterine cervix | NED(11) | Round to epithelioid | NA | Yes | *MALAT1-GLI1* |
|  | 4/M | Tongue | NED(11) | Round to epithelioid | NA | No | *GLI1* Amp |
|  | 75/F | Thigh | NED(9) | Round to epithelioid | NA | No | *GLI1-SYT* |
|  | 17/M | Tongue | NED(5) | Round to epithelioid | NA | No | *GLI1* Amp |
|  | 35/M | Groin | NED(4 | Round to epithelioid | NA | No | *NCOR2-GLI1* |
|  | 34/M | Mouth floor | Recent case | Round | NA | No | *MALAT1-GLI1* |
| Zhong HY et al | 56/M | Lingual | mets to LN and sacral(27)  AWD 36 mo | Round to epithelioid, myxoedematous stroma | 5/10HPF | focal | *GLI1* gene break-apart  Signals (FISH) |
| Yajuan J. Liu | 35/M | chest wall (multiple) | regional metastasis (involve a lymph node); NED(10) | ovoid to spindled cells | Rare | No | *TUBA1A-GLI1* |
| Natálie Klubíčková | 34/F | Oral cavity | NED(4) | Round to oval cells | 2/10HPF | No | *PTCH1-GLI1* |
| O.A. Abdelsadek | 53/M | abdomen | NA | Round to epithelioid cells | NA | NA | *ACTB-GLI1* |
| NR Godse | 54/F | left eye | NED(6) | spindled and epithelioid  cells | Rare | No | *GLI1* Amp |
| Ching-Ying Wang | 40/F | cervical spinal | NA | round to ovoid | 0/10HPF | No | *PTCH1-GLI1 ；GLI1-KDM2B* |
| Argani P et al | 33/F | renal pelvis | LR(25) | Ovoid-spindle cells | <1/10HPF | No | *GLI1* gene break-apart  Signals (FISH) |
|  | 49/F | Uterine | LR(24) and mets to brain(29). DOD 36 mo | Round to epithelioid (met) and spindled (LR) | 16/10HPF | Yes | *GLI1* Amp |
|  | 88/F | Uterine | involving the rectal adventitia | ovoid-spindle cell, myxoid stroma | 15/10HPF | focal | *GLI1* Amp |
|  | 49/F | Kidney | NED（84） | Ovoid-spindle cells | <1/10HPF | No | *GLI1-FOXO4* |
| Punjabi LS et al | 57/F | Uterine | LR, mets to LN and lung(11).  DOD 18 mo | Sarcomatous; pleomorphic cell | 40/10HPF | Yes | *PAMR1-GLI1* |
| Kerr DA et al | 40/F | Right thigh | bone and soft tissue met (77), ANED (89) | Ovoid-epithelioid cells, myxohyaline stroma | 6/2mm^2^ | No | *ACTB-GLI1* |
|  | 71/M | NA | lung met (bilateral), AWD (16) | ovoid-epithelioid cells,  myxoid stroma | 1/2mm^2^ | No | *ACTB-GLI1* |
|  | 31/M | Right leg | bone met(12), lung met (54), AWD (54) | ovoid-epithelioid cells, myxoidto collagenous stroma | 6/2mm^2^ | No | *ACTB-GLI1* |
|  | 56/M | Thoracic  vertebra T9 | recurrence(18), AWD (21) | ovoid-epithelioid cells, myxoid to collagenous stroma | ＜1/2mm^2^ | No | *ACTB-GLI1* |
|  | 26/F | T8-T9  Epidural  Tumor | LR(12, 105), lung met(36, 105), AWD(108) | Ovoid cells, myxoid stroma | 2/2mm^2^ | No | *ACTB-GLI1* |
|  | 46/M | Right pelvis  soft tissue | LR | Small ovoid-to-round  cells, solid to sieve-like, myxoid stroma | 1/2mm^2^ | No | *ACTB-GLI1* |
|  | 30/M | NA | lung met | ovoid-to-epithelioid cells, pseudoglandular,  myxoid matrix | ＜1/2mm^2^ | No | *ACTB-GLI1* |
|  | 37/F | Right neck | LR | ovoid-to-epithelioid cells, corded, myxoid stroma | 2/2mm^2^ | No | *PTCH1-GLI1* |
|  | 40/F | triceps | NED (7) | ovoid-to-round cells，myxohyaline stroma | ＜1/2mm^2^ | No | *ACTB-GLI1* |
|  | 22/F | Liver | NA | ovoid-to-spindle cells, pale myxoid stroma | 1/2mm^2^ | No | *ACTB-GLI1* |
|  | 60/M | Gluteus  maximus | NED (16) | ovoid-to-epithelioid cells;  myxohyaline stroma | 1/2mm^2^ | No | *ACTB-GLI1* |
|  | 51/F | Right neck  soft tissue | NED (12) | ovoid-to-spindle cells | ＜1/2mm^2^ | No | *ACTB-GLI1* |
|  | 2/M | Tongue | NA | ovoid-to-spindle cells | 8/2mm^2^ | No | *ACTB-GLI1* |
|  | 59/F | Right leg | NA | ovoid-to-epithelioid cells | 5/2mm^2^ | No | *ACTB-GLI1* |
|  | 60/M | Right  buttock | NA | ovoid-to-epithelioid cells | 1/2mm^2^ | No | *ACTB-GLI1* |
|  | 62/M | Left hard  palate | NA | ovoid-to-epithelioid cells | 1/2mm^2^ | No | *ACTB-GLI1* |
| Machado I et al | 65/F | left knee subcutaneous | skin of left tibial(1)；NED(8) | ovoid-epithelioid and spindled， microcystic | 2/10HPF | focal | *GLI1* Amp |
|  | 27/M | left hypothenar | NED(96) | round, epithelioid to ovoid cells | 3/10HPF | NA | *GLI1* Amp |
|  | 66/M | chest | Recurrence; NED(1) | Ovoid cells, focal pleomorphism | 8/10HPF | No | *GLI1* Amp |
| José Jessurun | 55/M | Terminal ileum | NED(36) | round or ovoid cells | 2/0.78mm^2^ | focal | *DDIT3-GLI1* |
|  | 22/F | Duodenum | NED(144) | ovoid to short spindled cells | 2/0.78mm^2^ | No | *GLI1* gene break-apart  Signals (FISH) |
|  | 80/F | Jejunum | NED(12) | round or ovoid cells | 2/0.78mm^2^ | No | *ACTB-GLI1* |
| Carina A | Young/M | tongue | NA | epithelioid cells | NA | NA | *ACTB-GLI1* |
| Neda Rezaee | 42/M | left postauricular scalp | NED(23) | epithelioid cells | Rare | No | *ATP2B4-GLI1* |
| Ying Zeng | 31/M | duodenal bulb | NED(6) | round to epithelioid cells | 1-2/10HPF | No | *ACTB-GLI1* |
| Jason R. Pettus | 55/M | left renal | NED(10) | spindle to epithelioid cells | Rare | No | *GLI1-FOXO4* |
| Jenny Lik-Ka Tse | 13/M | tongue | NED(6) | epithelioid to ovoid cells | 7/10HPF | No | *GLI1* Amp |
| Our case | 34/F | pleura | NED(15) | round to ovoid cells | ＜1/2mm^2^ | No | *PTCH1-GLI1* |

F, female; M, male; NA, not available; FU, follow up; mo, months; LR, local recurrence; LN, lymph node; met, metastasis; AWD, alive with disease; NED, no evidence; Amp, amplification
